# Void spot assay visualization optimization for use of Void Whizzard in rats (*Rattus norvegicus*)

**DOI:** 10.14814/phy2.70358

**Published:** 2025-05-05

**Authors:** Hannah Ruetten, Jadyn Bothe, Shannon Lankford, Gopal Badlani, James Koudy Williams

**Affiliations:** ^1^ Wake Forest Institute for Regenerative Medicine Wake Forest Baptist Medical Center Winston‐Salem North Carolina USA; ^2^ Department of Pathology, Section on Comparative Medicine Wake Forest School of Medicine Winston‐Salem North Carolina USA; ^3^ Department of Urology Wake Forest Baptist Medical Center Winston‐Salem Winston‐Salem North Carolina USA; ^4^ Section of Urology VA Medical Center Salisbury North Carolina USA

**Keywords:** lower urinary tract dysfunction, micturition, ninhydrin, urinary dysfunction, urine

## Abstract

Void spot assay (VSA) noninvasively evaluates urination. This study optimizes VSA by comparing post‐assay paper visualization techniques: bright field light (BF), ultraviolet light (UV), and ninhydrin spray (N). Male rats were placed in filter paper lined cages for 4 h. After the assay, all papers were dried. BF images were photographed (digital camera). UV images were captured using a Darkroom ultraviolet imaging cabinet. Papers were sprayed with ninhydrin and photographed (digital camera). All images were converted to binary for analysis with Void Whizzard. UV versus BF significantly differed in area. All three groups significantly differed in overall spot count and spots 0–0.1 cm^2^. UV versus N and UV versus BF significantly differed in 0.1–0.25 cm^2^ spots, UV versus N in 0.25–0.5 cm^2^, and N versus BF in spots 0.5–1 cm^2^. Overall BF visualization proved difficult. N provided an ideal way to highlight urine and image with a digital camera. Human fingerprints from pre‐assay handling of paper interfered with the analysis of the smallest sized spots; however, there were no differences in the detection of larger spots, spot distribution, or overall spot area. This study contributes to the development of a standardized VSA protocol for assessing bladder function in rodent models.

## 1 INTRODUCTION

A number of rat models exist for urinary disorders including bladder outlet obstruction, incontinence, pelvic floor dysfunction, spinal cord injury, and diabetic urinary dysfunction (Doelman et al., 2023; Fang et al., 2023; Shen et al., 2021). Rats are often preferred over mice when model creation or treatment interventions involve surgery due to their increased size. Many labs perform cystometry on rats or utilize metabolic cages to assess urinary function; however, these methods require expensive equipment and, at minimum, involve removing the rat from its home cage, which could impact urinary behavior due to handling stress and scent marking. In the case of cystometry, an invasive surgical procedure to implant a catheter in the bladder is performed, which impacts urinary function.

A common urinary function assessment performed in awake mice without any specialized equipment or surgical manipulation is the void spot assay. In the void spot assay, a mouse is placed in an enclosure lined with paper; they move freely while micturition is captured. The paper is then removed, imaged, and analyzed for some combination of spot size, spot count, and spot distribution. The assay in mice has been extensively optimized, software has been developed for quick and easy assessment, and the assay has been shown to have good predictability not only in showing changes in urinary function over time but also in discriminating differing voiding phenotypes from one another (Dalghi, 2021; Hill et al., 2018; Keil et al., 2016; Ruetten et al., 2021; Wegner et al., 2018).

Void spot assays are typically not performed in rats due to difficulty in analyzing papers from larger rat cages. However, some have performed “urine‐marking tests” in rats by hand drawing/tracing urine spots and performing manual measurements (Manzo et al., 2002). In a study by Wegner et al., it was shown that laboratory‐specific manual methods for void spot assay image analysis cause substantial variability in end point measurements, but this can be overcome by using a standardized analysis package (Wegner et al., 2018). Therefore, it would be ideal to optimize a way to image void spot assay papers from rats and use the images for analysis with Void Whizzard. This study aims to improve the feasibility of VSA for rats by testing agents to alter urine color and comparing post‐assay visualization techniques: bright field light, ultraviolet light, and ninhydrin spray.

## 2 MATERIALS AND METHODS

### 2.1 Rats

All experiments were conducted under an approved protocol from the Wake Forest University Animal Care and Use Committee and in accordance with the National Institutes of Health *Guide for the Care and Use of Laboratory Animals*. Heterogeneous stock rats (*n* = 32) were acquired as overstock from a campus breeding colony. Rats were housed in Allentown square microisolator rat caging on an Allentown rack (Allentown, Allentown, NJ). Room lighting was maintained on a 12:12‐h light–dark cycle, room temperature was typically 20.5 ± 5°C, and humidity was 30%–70%. Rats were fed 5P76 ‐ Prolab® IsoPro® RMH 3000, and food and water were available ad libitum. Cages contained Bed‐o'cob® ¼ bedding. All papers were collected at the baseline of the study from unmanipulated rats.

### 2.2 Color altering agents

Two rats were provided 200 mg of pharmaceutical grade prescription phenazopyridine (obtained from Atrium Health Wake Forest Baptist Pharmacy, Winston‐Salem, NC) dissolved in drinking water for 4 h, and water consumption was measured. The dose was halved to 100 mg and tested for the same duration in two other rats. Animal resources were contacted, and several combinations of phenazopyridine and sweeteners (honey, dextrose, and glucose) were trialed, and consumption was measured.

Rats were tested for consumption of beets and raspberries. Four rats were provided with two dishes per cage (1/rat) containing 4–6 cubes (5–15 mm at the largest dimension) of raw beetroot and 1 red raspberry at 2 pm the day prior to the void spot assay test. The test was repeated, providing the beets and raspberry at 5 pm the day prior to the void spot assay in two additional rats. Finally, all 32 rats were provided beets and a raspberry at 5 pm the day prior to the void spot assay test.

### 2.3 Spotting known volumes of urine

Free catch urine from female adult Sprague–Dawley rats was donated by a Wake Forest University Animal Care handling trainer for use in this project. Known volumes (5, 25, 50, and 100 microliters) were pipetted onto the four corners and center of the paper in a non‐overlapping manner. An additional 500 microliter volume was spotted in the center of the paper in a non‐overlapping manner. The paper was allowed to dry and then analyzed.

### 2.4 Void spot assay

We followed the recommended guidelines of reporting VSA data (Hill et al., 2018; Keil et al., 2016; Wegner et al., 2018). VSA was performed in the vivarium on the rack where the rats were housed. Cytiva Whatman™ 3MM Chr Chromatography Paper (26 × 41 cm sheets, pk 100) (No. 05‐713‐336, Fisher Scientific) were purchased and cut with a paper cutter to match the floor dimensions of the rats' home cage (26 × 36 cm). The paper was placed in the bottom of the cage with a nylabone placed in the center to discourage chewing. Rats were placed in the cage (singly housed) with food and water ad libitum for 4 h starting from 9 to 10 am Eastern time.

### 2.5 Ultraviolet light paper imaging

**FIGURE 1:**
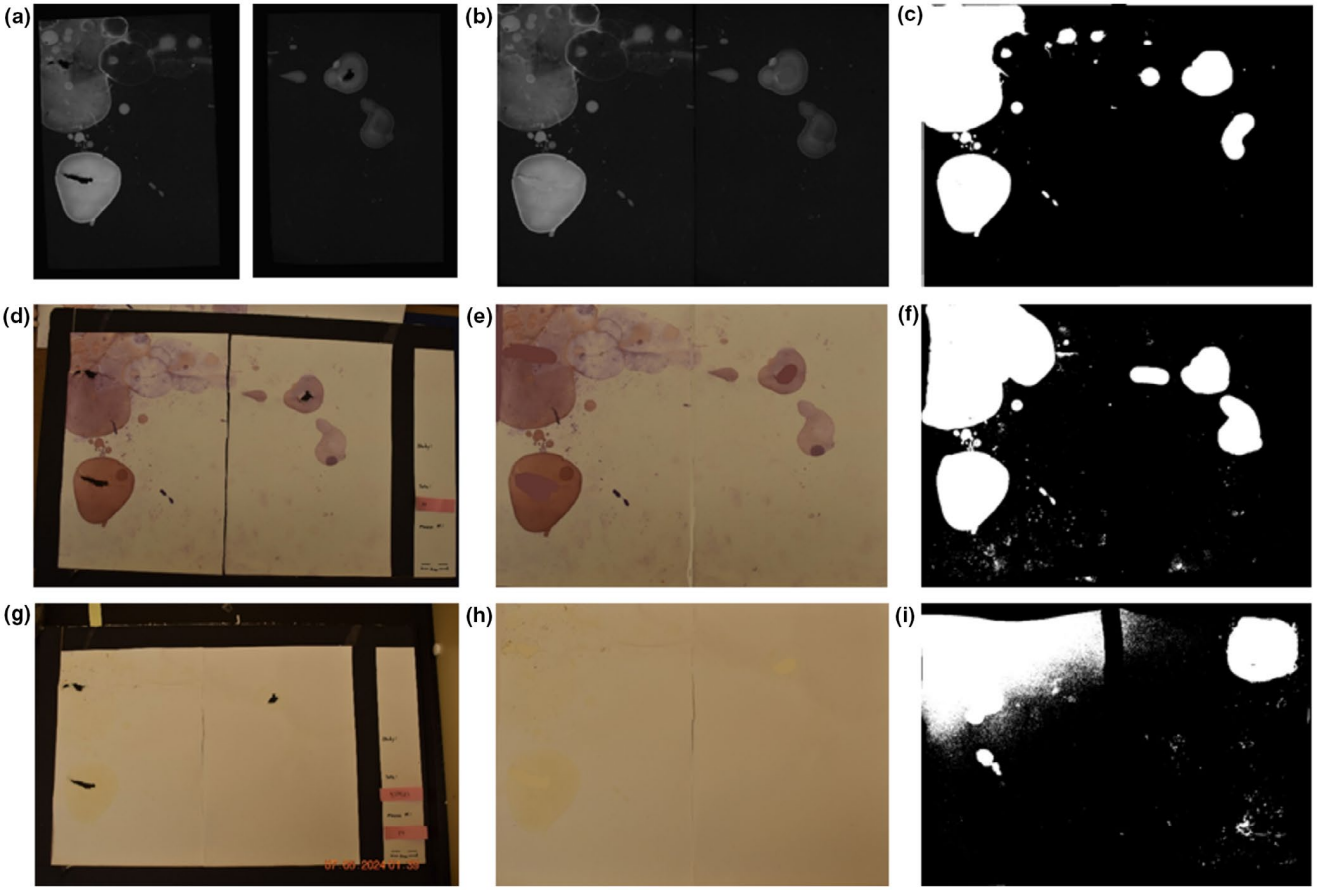
A comparison of the three imaging methods. (a) Ultraviolet light raw images from Autochemi AC1 Darkroom ultraviolet imaging cabinet, (b) raw images combined together, and (c) final black and white image for void spot assay analysis. (d) Picture of ninhydrin sprayed paper from digital camera, (e) image cropped and edited with chew spots filled in, and (f) final black and white image for void spot assay analysis. (g) Picture of void spot assay paper from digital camera, (h) image cropped and edited with chew spots filled in, and (i) final black and white image for void spot assay analysis.

Filter papers were dried, then cut in half and imaged with an Autochemi AC1 Darkroom ultraviolet imaging cabinet (UVP, Upland, CA) equipped with an Auto Chemi Zoom lens 2UV and an epi‐illuminator. Image capture settings were adjusted using UVP VisonWorksLS image acquisition software. Images were captured using an Ethidium Bromide filter set (570–640 nm) and 365‐nm epi‐illumination (Figure 1a). Raw .tif files of each half of the paper were recombined using GIMP 2.10.36 (revision 1). Any chewed areas were manually filled using white color with the paint brush in JS Paint version 1.0.0+ (Figure 1b). Final images were cropped to the paper boundaries, and an ImageJ macro was utilized (run(“16‐bit”); //run(“Brightness/Contrast…”); run(“Enhance Contrast”, “saturated = 0.35”); run(“Apply LUT”)) to enhance contrast and save as 16‐bit for analysis using Void Whizzard (Figure 1c).

### 2.6 Bright field paper imaging

An imaging board was constructed using black 3/16″ thick foam board composed of an extruded polystyrene core sandwiched between two layers of paper (Black Readi‐Board, R.L. Adams Plastics, Inc. Wyoming, MI). A large section of foam board was cut to a few inches larger than the VSA paper. Thin strips were then cut and layered on top at the left and bottom edge so there was a rigid L‐shaped ledge to place the paper on. On the right side of the board, a sheet of paper was placed with spaces to put the paper information to be recorded in the paper image. A scale bar was added so it was automatically included in the image. Papers were imaged with a Nikon D5600 digital camera (Figure 1d). Captured images were renamed with the paper information and cropped to the paper margins using GIMP 2.10.36 (revision 1) software. Any chewed areas were manually filled using light yellow color with the paint brush tool in JS Paint version 1.0.0+ (Figure 1e). Multiple techniques were trialed using ImageJ, including the threshold feature and the Color Deconvolution2 tool, to separate the urine spots from the white paper background. Utilizing the Color Deconvolution2 plugin, ROI vectors were selected, with the first color set to blue for optimal contrast, the second “color matched” to a urine spot, and the third color “color matched” to the paper. For thresholding, the process involved navigation to Image > Adjust > Color Threshold. In the popup window, the “dark background” option was deselected, and the default thresholding method, white threshold color, and HSB color space were used. The lighting conditions were slightly different between the initial imaging of known dotted volumes of urine and the analysis of actual void spot assay papers, so different threshold techniques were applied to detect the spots. For the known dotted volumes of urine, the following parameters were applied: Hue (select pass, 25–34), Saturation (select pass, 0–255), and Brightness (select pass, 0–197). For actual void spot assay papers, the following parameters were applied: Hue (pass deselect, 71–105), Saturation (select pass, 0–209), and Brightness (select pass, 0–97). Ultimately, the thresholding method was determined to be the most effective and used in final analysis for both use cases. Final images were cropped to the paper boundaries and converted to 16bit .tiff files for analysis using Void Whizzard (Figure 1f).

### 2.7 Ninhydrin paper imaging

Ninhydrin Aerosol Spray‐16 oz. (A‐2643, Thomas Scientific) was used to apply a light coat of spray to each urine paper. The papers were left to dry overnight. The same imaging board used for Bright field was used for Ninhydrin. Papers were imaged with a Nikon digital camera (Figure 1g). Captured images were relabeled with the paper information and cropped to the paper margins. Any chewed areas were manually filled using light purple color with the paint brush tool in JS Paint version 1.0.0+ (Figure 1h). Multiple techniques were trialed using ImageJ, including the threshold feature and Color Deconvolution2 tool, to separate the urine spots from the background white paper. For the Color Deconvolution2 plugin, ROI vectors were selected with the following parameters: the first color was set to yellow to achieve the greatest contrast against the purple ninhydrin spots, the second color was matched to a urine spot, and the third color was matched to the paper. The lighting conditions were slightly different between the initial imaging of known dotted volumes of urine and the analysis of actual void spot assay papers, so different threshold techniques were applied to detect the spots. For both thresholding techniques, (Image > Adjust > Color Threshold) in the popup window, “dark background” option was deselected, and the default thresholding method, black and white threshold color, and lab color space were used. For the known dotted volumes of urine, the following parameters were applied: L*(pass selected, 0–132), a* (pass selected, (134–147), and b* (pass selected, 0–255). For actual void spot assay papers, the following parameters were applied: L*(pass selected, 0–84), a* (pass selected, 0–255), and b* (pass selected, 0–255). The thresholding method was determined to be the most effective and used for final analysis. Final images were cropped to the paper boundaries and converted to 16bit .tiff files for analysis using Void Whizzard (Figure 1i).

### 2.8 Void paper analysis

Void Whizzard was downloaded from http://imagej.net/Void_Whizzard and run according to the user guide (Wegner et al., 2018). Analyzed parameters included total spot count, total void area (cm^2^), percent area in the center of the paper, percent area in corners of the paper, and mass distribution of spots (0–0.1, 0.1–0.25, 0.25–0.5, 0.5–1, 1–2, 2–3, 3–4, and 4+ cm^2^).

### 2.9 Statistical analysis

Statistical analyses were performed with Graph Pad Prism 8.0.2. Differences were considered significant at the *p* < 0.05 level. For group comparisons, a Kruskal–Wallis test was applied along with Dunn's multiple comparisons test.

## 3 RESULTS AND DISCUSSION

### 3.1 Rats would not drink water containing phenazopyridine even with a sweetener

Phenazopyridine (2,6‐diamino‐3‐[phenyl‐azo]pyridine) is used as an oral analgesic medication to treat patients with bladder inflammation. A universal side effect from taking this medication is that it turns the urine orange. Urologists, in addition to using the drug for treating bladder inflammation, have also leveraged this drug to color the urine orange, specifically for the purposes of cystoscopic identification of the ureteral orifices (Rehfuss et al., 2018). In this study, we attempted to color rat urine orange in order to allow for better bright field visualization of urine spots.

Rats were tested for consumption of phenazopyridine in drinking water. Two rats were provided 200 mg of phenazopyridine dissolved in drinking water for 4 h, and water consumption was measured. Neither rat consumed any water with phenazopyridine. They both immediately drank regular water upon returning it to the cage. The dose was halved to 100 mg and tested for the same duration in two other rats with the same results. Animal resources were contacted, and several combinations of phenazopyridine and sweeteners (honey, dextrose, and glucose) were trialed with no success in rats consuming the water.

In prior studies of phenazopyridine pharmacokinetics in rats, researchers dosed the medication via oral gavage (Chen et al., 2007). However, this would induce handling stress immediately prior to the void spot assay, so it was not pursued. Other researchers have dosed medications mixed into peanut butter and, anecdotally from a lab animal veterinary resident, rats seem to take medications well mixed into cream of chicken soup. Ultimately, these routes were also not pursued due to the need for handling and individual rat dosing.

If the phenazopyridine dosing had worked, there would also likely have been drug‐related effects that would have masked some urinary changes. When dosed intravenously in female Sprague–Dawley rats, phenazopyridine increases bladder compliance at 0.3, 1, and 3 mg/kg dose levels (Aizawa & Wyndaele, 2010).

### 3.2 Rats would consume beets and raspberries, but the resulting urine color alteration was not predictable and durable for the duration of the void spot assay

Beeturia occurs in 13.8% of the human population and is characterized by a pink to dark red discoloration of the urine (Watson et al., 1963). In rats dosed with fermented beet juice betacyanin derivatives, color changes are noted in the urine as soon as 15 min after administration and persist for at least 2 h (Sawicki et al., 2020). In rats dosed with beetroot powder, about 3% of the original betanin was recovered in the urine over the following 24 h (Krantz et al., 1980). Anecdotally, rats that receive a lot of beets or red berries in their diet can develop red discoloration of the urine that owners mistake for hematuria. However, beet overconsumption can lead to kidney stones (Mitchell et al., 2019) so beet intake must be kept within acceptable ranges set by animal resources. Based on this, we hypothesized that providing our rats with a moderate‐sized treat of beets and red raspberries the night before the void spot assay may discolor the urine pink to dark red and aid in void spot visualization.

Rats were tested for consumption of beets and raspberries. Four rats were provided two dishes per cage (1/rat) containing 4–6 cubes (5‐15 mm at the largest dimension) of raw beetroot and 1 red raspberry at 2 pm the day prior to the void spot assay test. Rats readily consumed the beets and raspberries. The void spot assay was performed the following morning at 9 am, and the urine took on a slight pink hue in a few voids for the rats but returned to normal color by the end of the assay. The test was repeated, providing the beets and raspberry at 5 pm the day prior to the void spot assay in two additional rats with similar results. All 32 rats were provided beets and a raspberry at 5 pm the day prior to the void spot assay test. Some voids had a slight pink hue but quickly faded to normal urine color before the papers could be analyzed.

The fading of the pink urine hue during paper drying is likely related to it becoming more alkaline. This also occurs with human urine samples which are darker pink when they are acidic, but the discoloration goes away as they become alkaline (Watson et al., 1963). Overall, the rats enjoyed their treat, but it did not provide the desired result of aiding in void bright field visualization.

### 3.3 Papers analyzed with the ninhydrin method have a similar Total void area, spot distribution, and number of larger spots to the ultraviolet light method

Since we were unable to color the urine in the rats, we next tested a method to color the urine after it was deposited on the paper. In a study, Arakawa et al. (2008) used Ninhydrin spray to detect urine from mouse scent marking assays. Ninhydrin is one of the most popular and widely used reagents for detecting proteins. It is used in a range of protein detection reactions, but one of its most commonly known uses is detection of fingerprints. Because of its strong color changing power in the presence of protein, it readily changes urine from a light yellow to a vibrant purple color. Here we compare visualization with ninhydrin to both ultraviolet light urine imaging and bright field imaging without any color altering aid. Full results are listed in Table 1.

**TABLE 1:** Void spot assay results summary.

Parameter	Ultraviolet (UV) *n* = 32 (mean ± SD)	Ninhydrin (N) *n* = 32 (mean ± SD)	Brightfield (BF) *n* = 32 (mean ± SD)	UV versus N (*p*‐value)	UV versus BF (*p*‐value)	N versus BF (*p*‐value)
Total void area	192.1 ± 93.99	166.2 ± 78.49	363.5 ± 288.7	>0.9999	**0.0011**	**<0.0001**
Total spot count	108.7 ± 92.56	751.0 ± 434.5	2103 ± 1242	**<0.0001**	**<0.0001**	**0.0030**
% center	22.57 ± 14.44	21.08 ± 14.76	23.32 ± 13.41	>0.9999	>0.9999	>0.9999
% corners	30.85 ± 11.11	33.57 ± 13.28	22.74 ± 12.30	>0.9999	**0.0263**	**0.0019**
0–0.1 cm^2^ spots	67.81 ± 72.52	678.4 ± 402.7	2046 ± 1215	**<0.0001**	**<0.0001**	**0.0025**
0.1–0.25 cm^2^ spots	16.56 ± 12.77	35.25 ± 20.50	30.47 ± 18.74	**0.0002**	**0.0042**	>0.9999
0.25–0.5 cm^2^ spots	7.156 ± 5.292	12.97 ± 7.966	6.563 ± 5.242	**0.0072**	>0.9999	**0.0013**
0.5–1 cm^2^ spots	4.531 ± 3.152	7.781 ± 5.326	3.219 ± 3.003	0.0737	0.2825	**0.0003**
1–2 cm^2^ spots	3.313 ± 3.167	5.969 ± 4.115	3.250 ± 3.742	**0.0164**	>0.9999	**0.0026**
2–3 cm^2^ spots	1.875 ± 1.641	2.875 ± 2.044	2.719 ± 2.986	0.1329	>0.9999	0.7185
3–4 cm^2^ spots	1.156 ± 1.194	1.094 ± 1.058	2.125 ± 1.930	>0.9999	0.1436	0.1468
4+ cm^2^ spots	6.313 ± 2.788	6.594 ± 4.047	8.375 ± 4.730	>0.9999	0.2141	0.3563

*Note*: Conversion of microliters of urine to spot area is approximately 11.4 μL per cm^2^ (range 9.6–14.7 μL/cm^2^). Comparisons with a p‐value <0.05 were considered statistically significant and are in bold.

Visually, ultraviolet images showed urine spots as black and white on a dark gray background with excellent contrast (Figure 1a–c). Ninhydrin produced spots that ranged in color from red‐orange to dark purple that were easily segregated from the background of the paper (Figure 1d–f). Bright field images had pale yellow urine spots with very little contrast to the background of the paper, making it difficult to write a macro that could distinguish urine spots from the background and from any dust or shadows in the image, as a result, dust and shadows were often detected as spots and falsely elevated spot counts (Figure 1g–i). All images were converted to binary, so there was no influence of spot intensity on analysis.

**FIGURE 2:**
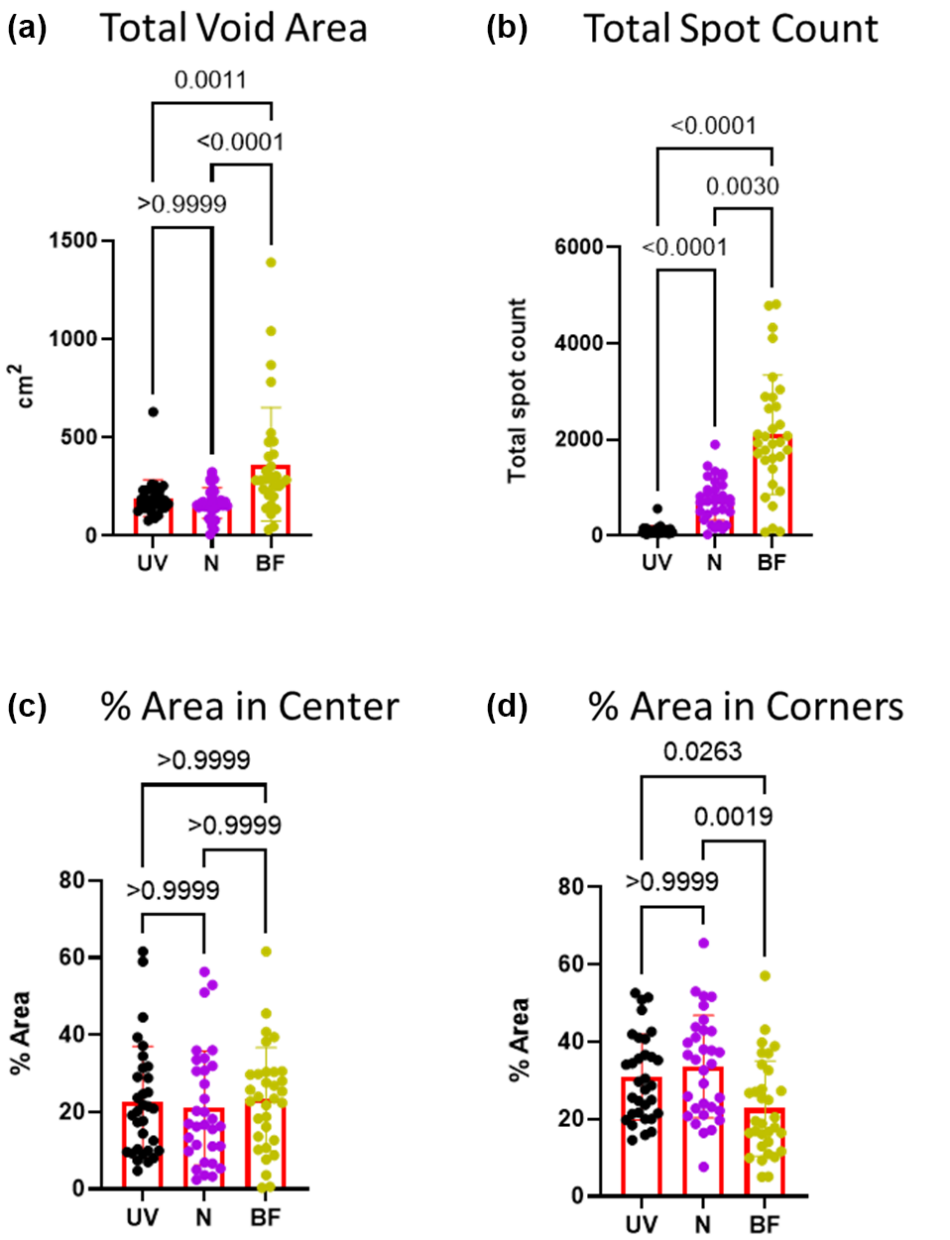
Papers analyzed with the ninhydrin method have a similar total void area and spot distribution to ultraviolet light method. Male rats (*n* = 32) were placed in cages lined by filter paper for 4 h. Water and food was provided ad lib. After the assay all papers were dried overnight. Bright field (BF) images were photographed using a digital camera. Ultraviolet light (UV) images were captured after cutting papers in half using a Darkroom ultraviolet imaging cabinet. Papers were sprayed with ninhydrin (N) and photographed (digital camera). All images were converted to black and white for analysis with Void Whizzard. Statistical analyses were performed with Graph Pad Prism 8.0.2. Differences were considered significant at the *p* < 0.05 level. For group comparisons, a Kruskal–Wallis test was applied along with Dunn's multiple comparisons test.

The first set of parameters we compared were total void area, total void count, percent void area in center, and percent void area in corners. The ninhydrin method had a similar total void area (*p* > 0.9999) (Figure 2a), percent void area in center (*p* > 0.9999) (Figure 2c), and percent void area in corner (*p* > 0.9999) (Figure 2d) compared to the ultraviolet method. The bright field method had a higher total void area (*p* = 0.0011, *p* < 0.0001) (Figure 2a), a similar percent void area in center (*p* > 0.9999, *p* > 0.9999) (Figure 2c), and decreased void area in corners (*p* = 0.0263, *p* = 0.0019) (Figure 2d) compared to the ultraviolet and ninhydrin methods. Total spot count was different for each method; it was slightly higher for ninhydrin (*p* < 0.0001) and much higher for the bright field (*p* < 0.0001) compared to the ultraviolet method (Figure 2b).

**FIGURE 3:**
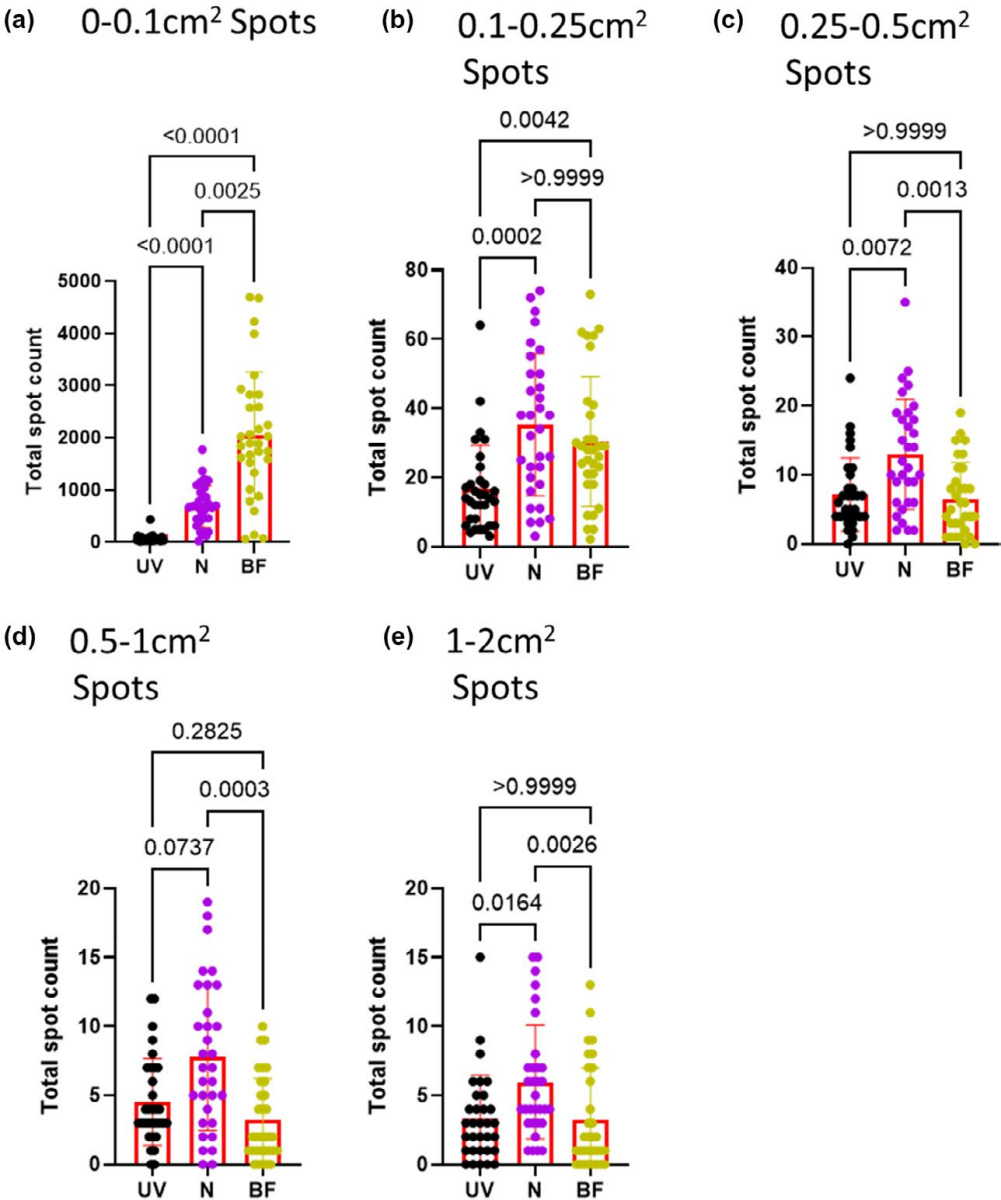
Papers analyzed with the ninhydrin method have more small spots compared to ultraviolet light method. Male rats (*n* = 32) were placed in cages lined by filter paper for 4 h. Water and food was provided ad lib. After the assay all papers were dried overnight. Bright field (BF) images were photographed using a digital camera. Ultraviolet light (UV) images were captured after cutting papers in half using a Darkroom ultraviolet imaging cabinet. Papers were sprayed with ninhydrin (N) and photographed (digital camera). All images were converted to black and white for analysis with Void Whizzard. Statistical analyses were performed with Graph Pad Prism 8.0.2. Differences were considered significant at the *p* < 0.05 level. For group comparisons, a Kruskal–Wallis test was applied along with Dunn's multiple comparisons test.

The next set of parameters we compared were the small spots: 0–0.1cm^2^ spots (~0‐1 μL), 0.1–0.25cm^2^ spots (~1.1–2.8 μL), 0.25–0.5cm^2^ spots (~2.9–5.6 μL), 0.5‐1 cm^2^ spots (~5.7–11.3 μL), and 1‐2 cm^2^ spots (~11.4–22.7 μL). The number of 0–0.1cm^2^ spots was different for each method; it was slightly higher for ninhydrin (*p* < 0.0001) and much higher for bright field (*p* < 0.0001) compared to ultraviolet method (Figure 3a). Both ninhydrin (*p* = 0.0002) and bright field (*p* = 0.0042) had more 0.1–0.25 cm^2^ spots than ultraviolet method (Figure 3b). For the ninhydrin method, we believe these increases in tiny spots were due to small rat footprints being detected as small urine spots. For bright field method, we believe these increases in tiny spots were due to shadow artifact and dust on the paper being detected as small urine spots. The number of 0.25–0.5cm^2^ spots was slightly higher for ninhydrin than bright field method (*p* = 0.0013) and ultraviolet method (*p* = 0.0072); bright field and ultraviolet were similar (*p* > 0.9999) (Figure 3c). The number of 0.5–1 cm^2^ spots was similar between ultraviolet method and ninhydrin method (*p* = 0.0737); bright field method was similar to ultraviolet method (*p* = 0.2825) but lower than ninhydrin (*p* = 0.0003) (Figure 3d). The number of 1–2 cm^2^ spots was slightly higher for ninhydrin than bright field method (*p* = 0.0026) and ultraviolet method (*p* = 0.0164); bright field and ultraviolet were similar (*p* > 0.9999) (Figure 3e). We believe these increases in 0.25–0.5 cm^2^ and 1–2 cm spots in ninhydrin compared to ultraviolet were due to partial and whole human fingerprints being detected as urine spots.

**FIGURE 4:**
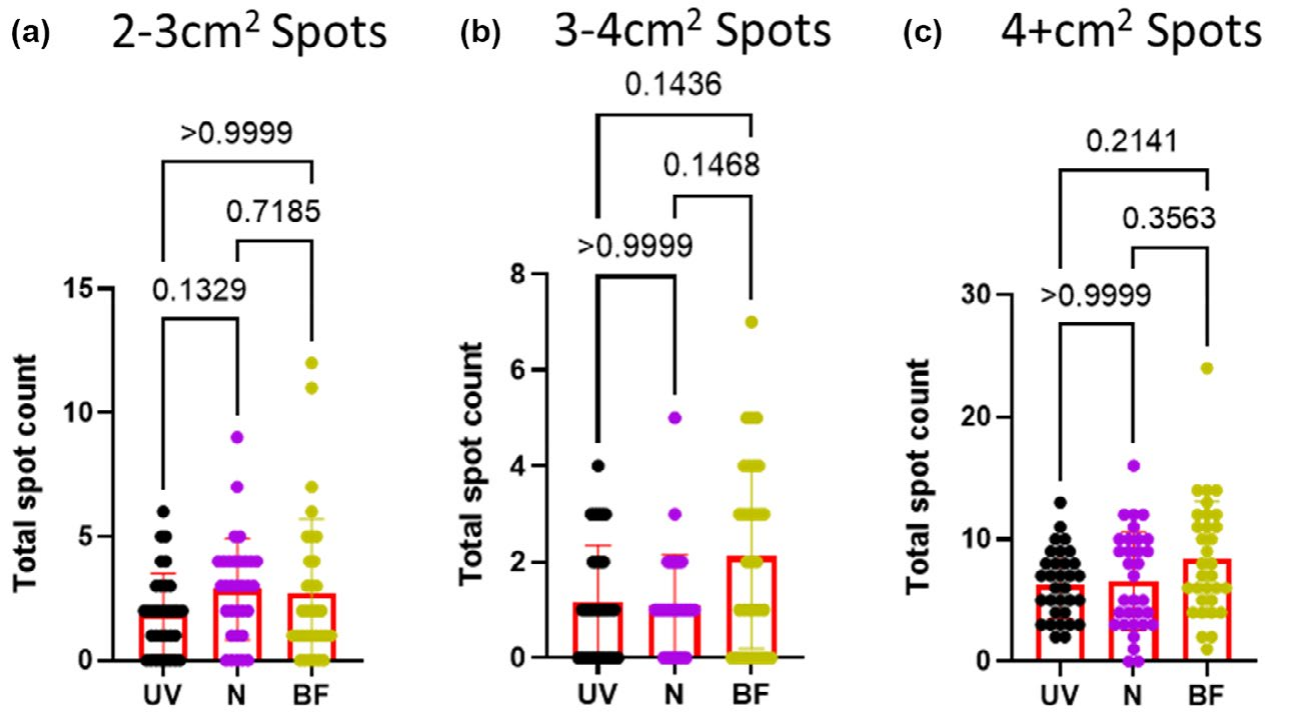
All three methods have similar numbers of large spots. Male rats (*n* = 32) were placed in cages lined by filter paper for 4 h. Water and food was provided ad lib. After the assay all papers were dried overnight. Bright field (BF) images were photographed using a digital camera. Ultraviolet light (UV) images were captured after cutting papers in half using a Darkroom ultraviolet imaging cabinet. Papers were sprayed with ninhydrin (N) and photographed (digital camera). All images were converted to black and white for analysis with Void Whizzard. Statistical analyses were performed with Graph Pad Prism 8.0.2. Differences were considered significant at the *p* < 0.05 level. For group comparisons, a Kruskal–Wallis test was applied along with Dunn's multiple comparisons test.

The final set of parameters we compared were the large spots: 2–3 cm^2^ spots (~22.8–34.1 μL), 3‐4 cm^2^ spots (~34.2–45.5 μL), and 4+ cm^2^ (>~45.6 μL). For all three spot sizes, the number of spots detected was similar among all three methods (*p* = 0.1329– >0.9999) (Figure 4).

**FIGURE 5:**
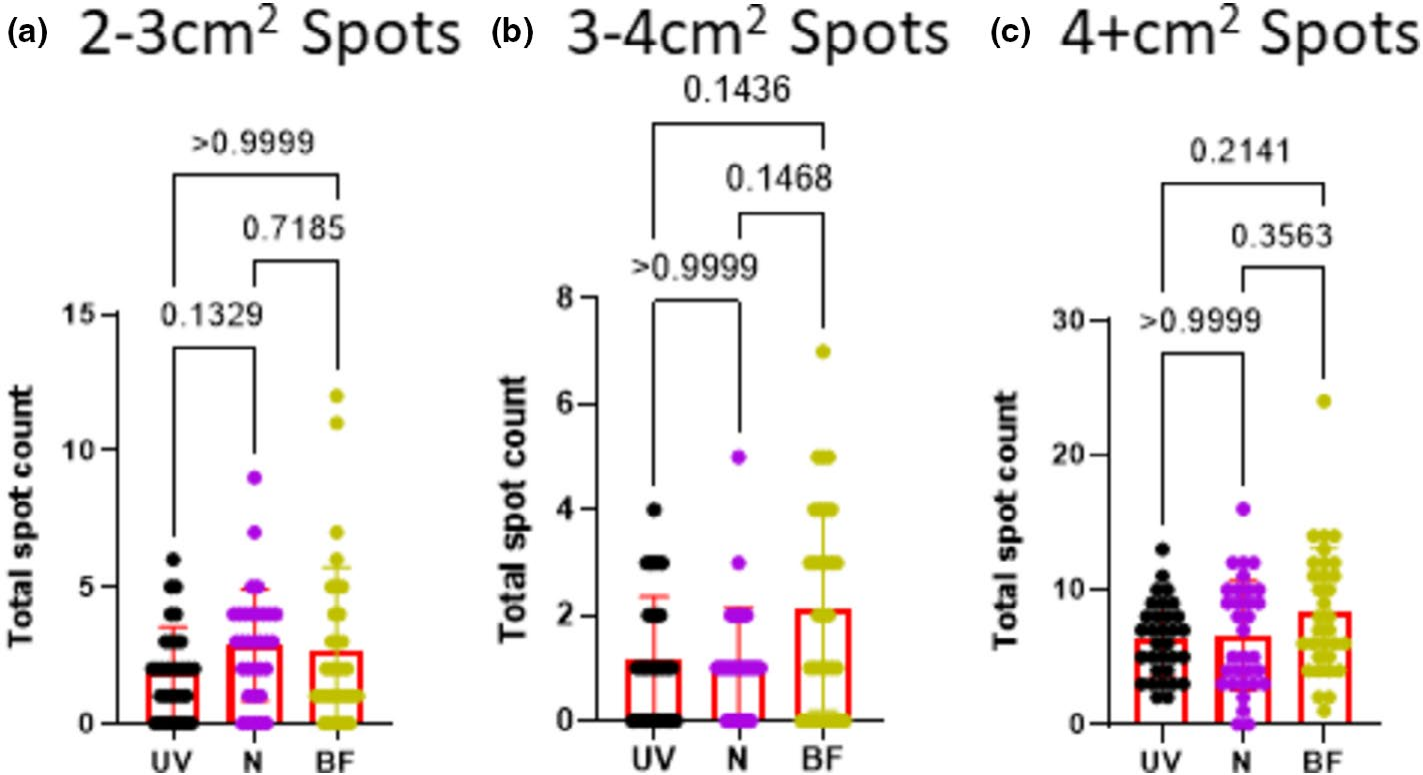
Ninhydrin provides more accurate detection of known urine spot volumes than brightfield. Urine from rats was manually pipetted as discrete spots in the corners and center of void spot assay paper. Five spots each containing 5, 25, 50, and 100 microliters of urine were spotted in both the corners and center of the paper. A single spot of 500 microliters of urine was spotted in the center of the paper. (a) Picture of cropped ninhydrin sprayed paper and (b) final black and white image for Void Whizzard analysis. (c) Picture of cropped brightfield paper and (d) final black and white image for Void Whizzard analysis. Each image is generated from the same 41 × 26 cm paper.

Because of the large discrepancy between brightfield, ninhydrin, and ultraviolet total spot counts and counts of smaller spot sizes, we performed follow‐up analysis on a test paper with known urine spot volumes to determine what was accounting for the spot count variation. Care was taken to avoid getting any fingerprints on the paper. Volumes of 5, 25, 50, 100, and 500 microliters (single spot, center only) of urine were pipetted onto the center and corners of void spot assay paper. The papers were then imaged and analyzed with the same techniques used for the true void spot assay papers. In total, 21 spots were placed on the paper. Using the ninhydrin method, the spots were easily visualized (Figure 5a) and detected (Figure 5b). With the brightfield method, the spots were hard to distinguish from the background (Figure 5c) and detection of discrete spots was difficult, with many portions of spots and background shadows being detected as small spots (Figure 5d). Void Whizzard detected 24 spots instead of 21 with the ninhydrin method due to three pinpoint‐sized white pixels being detected as the smallest spot size (0–0.1 cm^2^). The remaining 21 spots were accurately binned into the appropriate spot size (Table 2). Void Whizzard detected 1989 spots instead of 21 with the brightfield method due to the inaccurate detection of 1976 small spots (0–0.5 cm^2^) that were shadows or portions of the larger spots. Only 13 of the 21 true spots were detected, and many were inaccurately binned as smaller spots than what was spotted on the paper (Table 2). The conversion of microliters of urine to spot area was approximately 11.4 μL per cm^2^ (range 9.6–14.7 μL/cm^2^).

**TABLE 2:** Spotting known urine volumes results summary.

Parameter	Manual measurement	Ninhydrin (N)	Brightfield (BF)
Total void area (cm^2^)	110.1	128.6	79.0
Total spot count	21	24	1989
% center	44.4	43.9	47.9
% corners	55.6	55.5	41.0
0–0.1 cm^2^ spots	0	3	1908
0.1–0.25 cm^2^ spots	0	0	52
0.25–0.5 cm^2^ spots	0	0	16
0.5–1 cm^2^ spots	5	5	5
1–2 cm^2^ spots	0	0	2
2–3 cm^2^ spots	4	4	2
3–4 cm^2^ spots	1	1	2
4+ cm^2^ spots	11	11	2

A limitation of this study is that it solely focuses on optimizing the visualization of the paper itself and utilizes an existing analysis package rather than creating an analysis package for each imaging method. It is possible that “Void Whizzard” is better with ultraviolet images, and if we created a complete package including new analysis software for bright field and/or ninhydrin, we may have been able to further optimize the methods to make them more comparable to ultraviolet. However, the creation of a new software package was not within the scope of this study. In addition, a comparison of our void spot assays in rats to another urinary function analysis method may have provided additional optimization information; however, even in mice where the void spot assay is well established, void spot assay results and cystometry results often do not correlate.

## 4 CONCLUSIONS

When researchers have access to the more specialized equipment, the UV method remains the superior method for imaging void spot assay papers. However, due to potential exposure of humans to high levels of UV when imaging these large papers using investigator‐designed boxes (often void of safety features), the expense of commercial equipment able to accommodate large papers, and the added time to cut apart, image, and rebuild full images of the papers when using small gel imagers, ninhydrin provided an ideal alternative. Ninhydrin spray is relatively inexpensive and allows imaging of the papers with any camera. Human fingerprints from pre‐assay handling of paper as well as rat footprints interfered with the analysis of the smallest sized spots; however, human fingerprint interference is easily minimized by use of gloves when handling the paper pre‐ and post‐assay. Additionally, with the ninhydrin method, there were no differences in detection of the larger spots, spot distribution, or overall spot area. Brightfield imaging does not have adequate contrast between the pale yellow urine and the white paper to allow for accurate detection of spots. This study contributes to the development of a standardized VSA protocol for assessing bladder function in both mouse and rat models.

## AUTHOR CONTRIBUTIONS

Conceived and designed research (H.R.), performed experiments (H.R., S.S.L., J.B.), analyzed data (J.B. and H.R.), interpreted results of experiments (J.B. and H.R.), prepared figures (J.B. and H.R.), drafted manuscript (H.R.), edited and revised manuscript (H.R., J.B., S.S.L., G.B., and J.K.W.), approved final version of manuscript (H.R., J.B., S.S.L., G.B., and J.K.W.).

## FUNDING INFORMATION

Bothe supported by NIH R25 DK121572; Ruetten supported by NIH T32 OD010957; Research supported by a grant from The Veterans Association Merit Award.

## CONFLICT OF INTEREST STATEMENT

No conflicts of interest, financial or otherwise, are declared by the author(s).

## DISCLAIMERS

The funding sources had no influence over study design, execution, interpretations, or development of the manuscript. The contents do not represent the views of the U.S. Department of Veterans Affairs or the United States Government.

## ETHICS STATEMENT

All experiments were conducted under an approved protocol from the Wake Forest University Animal Care and Use Committee and in accordance with the National Institutes of Health *Guide for the Care and Use of Laboratory Animals*.

## Data Availability

Data will be made available upon request.
